# Immune checkpoint inhibitor-associated acute kidney injury and mortality: An observational study

**DOI:** 10.1371/journal.pone.0252978

**Published:** 2021-06-08

**Authors:** Marije S. Koks, Gurbey Ocak, Britt B. M. Suelmann, Cornelia A. R. Hulsbergen-Veelken, Saskia Haitjema, Marieke E. Vianen, Marianne C. Verhaar, Karin A. H. Kaasjager, Meriem Khairoun

**Affiliations:** 1 Department of Nephrology and Hypertension, University Medical Center Utrecht, Utrecht University, Utrecht, the Netherlands; 2 Department of Clinical Epidemiology, Leiden University Medical Center, Leiden, the Netherlands; 3 Department of Internal Medicine, St. Antonius Hospital, Nieuwegein, the Netherlands; 4 Department of Medical Oncology, University Medical Center Utrecht, Utrecht University, Utrecht, the Netherlands; 5 Central Diagnostic Laboratory, University Medical Center Utrecht, Utrecht University, Utrecht, the Netherlands; 6 Department of Internal Medicine and Dermatology, University Medical Center Utrecht, Utrecht University, Utrecht, the Netherlands; University of Toledo, UNITED STATES

## Abstract

**Background:**

Immune checkpoint inhibitors, approved for the treatment of various types of cancer, are known to cause a unique spectrum of side effects, including acute kidney injury (AKI). The aim of this study was to describe the incidence, risk factors, renal outcomes, and mortality of AKI in patients receiving checkpoint inhibitors.

**Methods:**

Patients receiving checkpoint inhibitors between January 2013 and May 2020 at the University Medical Center Utrecht, the Netherlands, were identified using the Utrecht Patient Oriented Database. AKI was defined as an increase in serum creatinine of ≥1.5 times the baseline value, based on the Kidney Disease: Improving Global Outcomes criteria. Cox proportional hazard regression analysis was used to assess risk factors for AKI and to evaluate the relationship between AKI and mortality. Persistent renal dysfunction was diagnosed in AKI patients with a final serum creatinine measurement of >1.3 times the baseline value.

**Results:**

Among 676 patients receiving checkpoint inhibitors, the overall incidence of AKI was 14.2%. Baseline variables independently associated with AKI were a gynecologic malignancy, monotherapy with ipilimumab, and the use of a diuretic, angiotensin-converting enzyme inhibitor or angiotensin-receptor blocker, or proton pump inhibitor at baseline. AKI was checkpoint inhibitor-associated in one third of all patients with AKI. Checkpoint inhibitor-associated AKI was mostly low-grade, occurred a median of 15 weeks after checkpoint inhibitor initiation, and resulted in persistent renal dysfunction in approximately 40% of the patients. Patients with all-cause AKI had a twofold increased mortality risk, but checkpoint inhibitor-associated AKI was not associated with increased mortality.

**Conclusions:**

In this study, patients receiving checkpoint inhibitors frequently developed AKI due to various etiologies. AKI directly related to the effect of checkpoint inhibitor toxicity did not increase mortality. However, AKI not related to the effect of checkpoint inhibitor toxicity was associated with increased mortality.

## Introduction

In the last decade, immune checkpoint inhibition has become an important treatment for patients with a broad range of malignancies, including melanoma, non-small-cell lung carcinoma, urinary tract cancer, and Hodgkin lymphoma [1–5]. Immune checkpoint inhibitors (ICPi) are humanized monoclonal antibodies that increase antitumor immune responses by blocking inhibitory pathways in T lymphocytes [6,7]. The first ICPi, targeting cytotoxic T lymphocyte–associated antigen 4, earned FDA approval in 2011 [8]. ICPi blocking programmed cell death protein 1 and programmed death ligand 1 have followed since, and novel agents are currently entering clinical trials [9].

Alongside their beneficial effects in patients with cancer, ICPi are known to cause a unique spectrum of autoimmune-related side effects through unrestrained T cell activation. These immune-related adverse events (irAE) may affect multiple organ systems, typically involving the skin, gastrointestinal tract, liver, and endocrine system [10,11]. Renal dysfunction, presenting as acute kidney injury (AKI), is increasingly recognized as an irAE of ICPi [12]. Diagnosing ICPi-associated AKI (ICPi-AKI) can be challenging as patients with advanced malignancies may have various other reasons for developing AKI, including fluid depletion, infection, exposure to nephrotoxic agents, cardiac failure, hypercalcemia, or urinary tract obstruction due to tumor progression [13]. The majority of kidney biopsies in patients with suspected ICPi-AKI show acute tubulointerstitial nephritis as the dominant histopathological lesion, although glomerular diseases are also reported [14–17].

Recently, two observational studies showed that AKI is common in patients receiving ICPi, both reporting an incidence of 17% [18,19]. These studies also aimed to identify risk factors for development of AKI in ICPi users. The first and largest cohort study reported proton pump inhibitor use at baseline to be associated with increased risk of AKI, while the most recent cohort study found previous diagnosis of hypertension and development of non-kidney irAE to increase AKI risk. As the reported risk factors for AKI are inconsistent, further research on this topic is necessary. Other issues that require further investigation is whether development of ICPi-AKI is associated with long-term renal dysfunction and increased mortality. Insights regarding long-term renal function and mortality might affect decision-making on continuation of ICPi treatment in patients who develop ICPi-AKI and hence affect cancer outcomes.

The aim of this study was to describe the incidence, risk factors, and mortality of AKI in patients receiving ICPi. Furthermore, we sought to investigate renal outcomes after development of AKI in ICPi users.

## Materials and methods

### Study population

We performed a retrospective observational cohort study of patients aged ≥18 years who received ICPi between January 1, 2013, and May 31, 2020, at the University Medical Center Utrecht, an academic hospital in the Netherlands. Patients who received nivolumab, ipilimumab, pembrolizumab, atezolizumab, durvalumab, or tremelimumab were included. When patients were consecutively treated with more than one ICPi, i.e. 9.5% of all patients, only the first ICPi was recorded. We excluded patients on renal replacement therapy, without serum creatinine measurement in the 12 months prior to first ICPi administration, or without any serum creatinine measurement after first ICPi administration. This study was performed in accordance with the Declaration of Helsinki. The Medical Research Ethics Committee Utrecht decided that institutional review board approval was not required, as no patients were subjected to interventions nor were any rules of conduct imposed upon them. Patient IDs were visible to the researchers in order to access electronic patient files, required to determine presumptive AKI etiologies. All data were anonymized before analysis. Follow-up started at first ICPi administration and ended with either death or the end of follow-up (July 31, 2020), whichever occurred first.

### Data collection

Patients receiving ICPi were identified through the Utrecht Patient Oriented Database (UPOD). In brief, UPOD is an infrastructure of relational databases based on the hospital-wide electronic patient record systems, comprising characteristics of all patients treated at the University Medical Center Utrecht since 2004. The structure and content of UPOD have been described in more detail elsewhere [20]. Through UPOD, data on patient age, sex, malignancy type, comorbid conditions, date of death, serum creatinine measurements, and baseline use of antihypertensive- and nephrotoxic medication were collected. Treatment with potentially nephrotoxic chemotherapy or targeted therapy during the six months prior to first ICPi administration was recorded (Table 1). Patients were defined as having hypertension if they were using antihypertensive medication at baseline. The Charlson Comorbidity Index was used to quantify the extent of comorbidities, including diabetes, chronic kidney disease, and cardiovascular diseases [21]. As ICPi-associated myositis has been reported and would be accompanied with serum creatinine elevation not due to kidney dysfunction, episodes of myositis were recorded [22]. The estimated glomerular filtration rate (eGFR) was calculated using the Chronic Kidney Disease Epidemiology Collaboration equation [23].

**Table 1:** Baseline characteristics.

	All patients N = 676		No AKI N = 580		AKI N = 96	
Age, median (IQR)	64	(53–71)	64	(54–71)	64	(53–73)
Female (%)	256	(37.9)	215	(37.1)	41	(42.7)
eGFR (mL/min/1.73 m^2^), median (IQR)	90	(75–101)	90	(75–101)	92	(73–103)
eGFR group						
<60 mL/min/1.73 m^2^ (%)	68	(10.1)	57	(9.8)	11	(11.5)
60–90 mL/min/1.73 m^2^ (%)	263	(38.9)	229	(39.5)	34	(35.4)
≥90 mL/min/1.73 m^2^ (%)	345	(51.0)	294	(50.7)	51	(53.1)
Malignancy type						
Melanoma (%)	319	(47.2)	273	(47.1)	46	(47.9)
NSCLC (%)	163	(24.1)	143	(24.7)	20	(20.8)
Gynecologic cancer (%)	19	(2.8)	12	(2.1)	7	(7.3)
Urinary tract cancer (%)	81	(12.0)	72	(12.4)	9	(9.4)
Other (%)	94	(13.9)	80	(13.8)	14	(14.6)
Charlson Comorbidity Index, median (IQR)	8	(7–9)	8	(7–9)	8	(7–9)
Diabetes (%)	63	(9.3)	51	(8.8)	12	(12.5)
Hypertension (%)	234	(34.6)	196	(33.8)	38	(39.6)
Medication						
ACEi or ARB, diuretic, PPI, or NSAID (%)	344	(50.9)	284	(49.0)	60	(62.5)
ACEi or ARB (%)	147	(21.7)	119	(20.5)	28	(29.2)
Diuretic (%)	96	(14.2)	76	(13.1)	20	(20.8)
PPI (%)	243	(35.9)	200	(34.5)	43	(44.8)
NSAID (%)	64	(9.5)	53	(9.1)	11	(11.5)
Prior chemotherapy or targeted therapy^a^ (%)	193	(28.6)	162	(27.9)	31	(32.3)
Checkpoint inhibitor type						
Nivolumab (%)	202	(29.9)	176	(30.3)	26	(27.1)
Ipilimumab (%)	45	(6.7)	35	(6.0)	10	(10.4)
Pembrolizumab (%)	236	(34.9)	200	(34.5)	36	(37.5)
Combined therapy^b^ (%)	132	(19.5)	113	(19.5)	19	(19.8)
Other (%)	61	(9.0)	56	(9.7)	5	(5.2)

ACEi, angiotensin-converting enzyme inhibitor; AKI, acute kidney injury; ARB, angiotensin-receptor blocker; eGFR, estimated glomerular filtration rate; NSAID, non-steroidal anti-inflammatory drug; NSCLC, non-small-cell lung carcinoma; PPI, proton pump inhibitor.

^a^Chemotherapy or targeted therapy included carboplatin, cisplatin, oxaliplatin, gemcitabine, capecitabine, cyclophosphamide, methotrexate, pemetrexed, mitomycin, topotecan, ifosfamide, irinotecan, axitinib, imatinib, pazopanib, bortezomib, bevacizumab, sorafenib, sunitinib, bortezomib, carfilzomib, everolimus, temsirolimus, imatinib, dasatinib, vemurafenib, dabrafenib, cetuximab, panitumumab, venetoclax, lenalidomide, crizotinib, and ibrutinib.

^b^Combined therapy consisted of nivolumab and ipilimumab.

### Outcomes

The primary outcome was development of AKI, defined as an increase in serum creatinine of ≥1.5 times the baseline value, based on the Kidney Disease: Improving Global Outcomes (KDIGO) criteria [24]. Baseline creatinine was defined as the most recent creatinine measurement before the date of first ICPi administration (median of 1 (IQR 0 to 4) day, maximum of 37 days). The highest creatinine value within three months of AKI onset was used to define AKI as KDIGO stage 1, stage 2, and stage 3, referring to an increase of 1.5 to 1.9, 2.0 to 2.9, and ≥3.0 times the baseline creatinine value, respectively. We analyzed predetermined baseline variables as potential risk factors for AKI. Furthermore, we evaluated renal outcome (persistent renal dysfunction) and mortality in patients who had developed AKI and ICPi-AKI. Persistent renal dysfunction was diagnosed in AKI patients with a final creatinine measurement (at the end of follow-up) of >1.3 times the baseline value. This definition was based on results of a study comparing different definitions of renal recovery after cardiac surgery-associated AKI [25].

### ICPi-AKI versus non-ICPi-AKI

AKI etiology was determined by retrospective chart review performed by three researchers. A novel definition and classification system for ICPi-AKI, proposed by Gupta et al., was applied to distinguish ICPi-AKI from other AKI etiologies (non-ICPi-AKI) [26]. This classification acknowledges several gradations of diagnostic uncertainty in the absence of kidney biopsy by identifying definite, probable, and possible ICPi-AKI. Definite ICPi-AKI was confirmed when kidney biopsy showed a histopathological lesion compatible with nephrotoxicity caused by ICPi. Probable ICPi-AKI was allocated to patients meeting three criteria: 1) creatinine elevation of ≥1.5 times the baseline value on at least two consecutive values or need for renal replacement therapy; 2) absence of an alternative plausible cause; 3) at least one of three additional criteria: sterile pyuria, eosinophilia, or recent or concomitant non-kidney irAE. An AKI episode was labeled as possible ICPi-AKI when an alternative etiology was not readily attributable, i.e. there was no prior or simultaneous episode of dehydration, hypotension, sepsis, hypercalcemia, acute cardiac event, excessive bleeding, myeloma cast nephropathy, hydronephrosis, or bladder outlet obstruction. AKI was considered non-ICPi-related in patients with hemodynamic or pre-renal causes (including acute tubular necrosis), post-renal obstruction, or other causes.

### Statistical analysis

We present continuous variables with medians and interquartile ranges (IQRs) and categorical variables with counts and percentages. The association between baseline characteristics and AKI was evaluated using Cox proportional hazard regression analysis. Persistent renal dysfunction rates in patients with ICPi-AKI versus non-ICPi-AKI were compared using a Pearson’s chi-squared test. To evaluate the association between AKI and subsequent mortality, a time-dependent Cox proportional hazard regression model was used [27]. Crude and adjusted hazard ratios (HRs) with 95% confidence intervals (CIs) were calculated. HRs were adjusted for age, sex, baseline eGFR, malignancy type, hypertension, baseline exposure to a diuretic, angiotensin-converting enzyme inhibitor (ACE-inhibitor) or angiotensin-receptor blocker (ARB), proton pump inhibitor, or non-steroidal anti-inflammatory drug (NSAID), chemotherapy, checkpoint inhibitor type, and Charlson Comorbidity Index. All analyses were performed using IBM SPSS Statistics version 25.0.

## Results

Of the 678 patients who received ICPi between January 1, 2013, and May 31, 2020, one patient had previously objected to use of personal data for research, and one patient had no baseline creatinine measurement, resulting in inclusion of 676 patients in the cohort. Baseline characteristics of the 676 patients are shown in Table 1. The median age was 64 (IQR 53 to 71) years, 256 (37.9%) patients were female, and the median baseline eGFR was 90 (IQR 75 to 101) mL/min/1.73 m^2^. Melanoma (47.2%) was the most frequent malignancy type, followed by non-small-cell lung carcinoma (24.1%). Nivolumab was the most commonly prescribed ICPi when considering both monotherapy and combined therapy, used by 334 (49.4%) patients.

### Incidence and causes of AKI

A total of 96 (14.2%) patients developed AKI following ICPi therapy, during a total follow-up time of 856 person-years (Table 2). Stage 1 AKI occurred in 73 (76.0%), stage 2 in 16 (16.7%), and stage 3 in seven (7.3%) patients. None of the patients required renal replacement therapy. The rate of AKI was 112 per 1000 person-years. Median time from first ICPi administration to AKI was 15 (IQR 5 to 37) weeks. A nephrologist was consulted in 12 (12.5%) patients with AKI, and urinalysis was performed in 54 (56.3%) patients with AKI. Urinalysis showed leucocyturia in 20 patients (37.0% of all AKI patients with urinalysis) and microscopic hematuria in 18 patients (33.3% of all AKI patients with urinalysis). Among all patients meeting AKI criteria based on serum creatinine elevation (N = 96), one patient was diagnosed with myositis, however, this diagnosis was established more than six months after the initial serum creatinine elevation.

**Table 2:** Stage, etiology, and renal outcome of patients with AKI and ICPi-AKI.

**Patients with AKI**	**96**	**(14.2% of all patients)**
AKI stage		
Stage 1 (%)	73	(76.0)
Stage 2 (%)	16	(16.7)
Stage 3 (%)	7	(7.3)
Renal outcome after AKI		
Persisting renal dysfunction (%)	34	(35.4)
Renal recovery (%)	62	(64.6)
Etiology among patients with AKI		
Definite ICPi-AKI (%)	1	(1.0)
Probable ICPi-AKI (%)	8	(8.3)
Possible ICPi-AKI (%)	23	(24.0)
Hemodynamic or pre-renal cause (%)	53	(55.2)
Post-renal cause (%)	7	(7.3)
Other cause (%)	4	(4.2)
**Patients with ICPi-AKI**	**32**	**(4.7% of all patients)**
ICPi-AKI stage		
Stage 1 (%)	26	(81.3)
Stage 2 (%)	6	(18.8)
Stage 3 (%)	0	
Renal outcome after ICPi-AKI		
Persisting renal dysfunction (%)	13	(40.6)
Renal recovery (%)	19	(59.4)

AKI, acute kidney injury; ICPi-AKI, immune checkpoint inhibitor-associated acute kidney injury.

Chart review regarding causes of AKI in all 96 patients revealed that 32 patients (33.3% of all AKI patients) had ICPi-AKI, which was subdivided into three levels of diagnostic certainty. One (1.0%) patient was diagnosed with definite ICPi-AKI as confirmed by kidney biopsy, which showed acute tubulointerstitial nephritis. Eight (8.3%) patients met the criteria of probable ICPi-AKI, and 23 (24.0%) patients had possible ICPi-AKI. In 53 (55.2%) patients, AKI was likely related to a hemodynamic or pre-renal cause, and in seven (7.3%) patients, AKI was attributed to post-renal obstruction. Four (4.2%) patients had AKI due to other causes, including urinary tract infection and myeloma cast nephropathy.

Regarding the 32 patients with definite, probable, and possible ICPi-AKI, 81.3% had stage 1 AKI and 18.8% had stage 2 AKI. There were no cases of stage 3 AKI among patients with ICPi-AKI. Median time from first ICPi administration to ICPi-AKI was 15 (IQR 5 to 31) weeks. A nephrologist was consulted in six (18.8%) ICPi-AKI patients, and urinalysis was performed in 14 (43.8%) ICPi-AKI patients. Urinalysis showed leucocyturia in six patients (42.9% of all ICPi-AKI patients with urinalysis) and microscopic hematuria in three patients (21.4% of all ICPi-AKI patients with urinalysis). Corticosteroid treatment was started in 11 (34.4%) patients with ICPi-AKI and ICPi treatment was interrupted in 14 (43.8%) patients with ICPi-AKI. Three patients with ICPi-AKI were re-challenged with ICPi, whereupon one patient once more developed AKI. This AKI episode was attributed to a combination of ICPi nephrotoxicity and a hemodynamic cause.

### Risk factors for AKI

Analysis of the relationship between baseline variables and development of AKI showed that, after adjustment for potential confounders, a gynecologic malignancy was associated with a 3.91-fold (95% CI 1.55 to 9.85) increased risk of AKI, and monotherapy with ipilimumab was associated with a 2.31-fold (95% CI 1.03 to 5.20) increased risk of AKI. The risk of developing AKI was increased 2.61-fold (95% CI 1.21 to 5.60) with the use of a diuretic, 2.49-fold (95% CI 1.10 to 5.60) with the use of an ACE-inhibitor or ARB, and 1.69-fold (95% CI 1.04 to 2.75) with the use of a proton pump inhibitor (Table 3). Diabetes was not associated with AKI (crude HR 1.44, 95% CI 0.79 to 2.64; adjusted HR 1.06, 95% CI 0.55 to 2.05).

**Table 3:** Risk factors for AKI.

		**Hazard ratio (95% CI)**			
		**Crude**		**Adjusted**	
Age (years)*	<65	1	(reference)	1	(reference)
	≥65	0.97	(0.65–1.45)	0.86	(0.51–1.45)
Sex^†^	Male	1	(reference)	1	(reference)
	Female	1.21	(0.80–1.81)	1.24	(0.82–1.88)
Baseline eGFR group^‡^ (mL/min/1.73 m^2^)	≥90	1	(reference)	1	(reference)
	60–90	0.85	(0.55–1.31)	0.78	(0.48–1.27)
	<60	1.10	(0.57–2.11)	0.82	(0.40–1.68)
Malignancy type^§^	Melanoma	1	(reference)	1	(reference)
	NSCLC	0.91	(0.54–1.54)	0.67	(0.38–1.19)
	Gynecologic cancer	3.38	(1.52–7.51)	3.91	(1.55–9.85)
	Urinary tract cancer	0.93	(0.46–1.91)	0.93	(0.44–2.00)
	Other	1.09	(0.60–1.98)	1.28	(0.67–2.44)
Charlson Comorbidity Index^#^	0–3	1	(reference)	1	(reference)
	4–7	1.17	(0.36–3.83)	1.27	(0.36–4.56)
	8–11	1.36	(0.43–4.34)	1.67	(0.44–6.32)
	≥12	2.98	(0.67–13.33)	3.17	(0.56–17.97)
Hypertension^¶^	No	1	(reference)	1	(reference)
	Yes	1.30	(0.86–1.96)	0.66	(0.31–1.40)
Medication use at baseline^π^	No ACEi or ARB, diuretic, PPI or NSAID	1	(reference)	1	(reference)
	ACEi or ARB	1.99	(1.21–3.27)	2.49	(1.10–5.60)
	Diuretic	2.15	(1.25–3.72)	2.61	(1.21–5.60)
	PPI	1.86	(1.19–2.89)	1.69	(1.04–2.75)
	NSAID	1.81	(0.92–3.55)	1.63	(0.81–3.26)
Prior chemotherapy or targeted therapy^Δ^	No	1	(reference)	1	(reference)
	Yes	1.41	(0.92–2.16)	1.41	(0.90–2.21)
Checkpoint inhibitor type^■^	Nivolumab	1	(reference)	1	(reference)
	Ipilimumab	1.72	(0.83–3.57)	2.31	(1.03–5.20)
	Pembrolizumab	1.23	(0.74–2.03)	1.52	(0.89–2.60)
	Combined therapy^a^	1.26	(0.70–2.29)	1.72	(0.93–3.21)
	Other	0.63	(0.24–1.64)	0.49	(0.18–1.29)

ACEi, angiotensin-converting enzyme inhibitor; AKI, acute kidney injury; ARB, angiotensin-receptor blocker; eGFR, estimated glomerular filtration rate; NSAID, non-steroidal anti-inflammatory drug; NSCLC, non-small-cell lung carcinoma; PPI, proton pump inhibitor.

*Adjusted for sex, baseline eGFR, malignancy type, hypertension, baseline exposure to ACEi or ARB, diuretic, PPI, and NSAID, prior chemotherapy, checkpoint inhibitor type, and Charlson Comorbidity Index.

^†^Adjusted for age, baseline eGFR, malignancy type, hypertension, baseline exposure to ACEi or ARB, diuretic, PPI, or NSAID, prior chemotherapy, checkpoint inhibitor type, and Charlson Comorbidity Index.

^‡^Adjusted for age, sex, malignancy type, hypertension, baseline exposure to ACEi or ARB, diuretic, PPI, or NSAID, prior chemotherapy, checkpoint inhibitor type, and Charlson Comorbidity Index.

^§^Adjusted for age, sex, baseline eGFR, hypertension, baseline exposure to ACEi or ARB, diuretic, PPI, or NSAID, prior chemotherapy, checkpoint inhibitor type, and Charlson Comorbidity Index.

^#^Adjusted for age, sex, baseline eGFR, malignancy type, hypertension, baseline exposure to ACEi or ARB, diuretic, PPI, or NSAID, prior chemotherapy, and checkpoint inhibitor type.

^¶^Adjusted for age, sex, baseline eGFR, malignancy type, baseline exposure to ACEi or ARB, diuretic, PPI, or NSAID, prior chemotherapy, checkpoint inhibitor type, and Charlson Comorbidity Index.

^π^Adjusted for age, sex, baseline eGFR, malignancy type, hypertension, prior chemotherapy, checkpoint inhibitor type, and Charlson Comorbidity Index.

^Δ^Adjusted for age, sex, baseline eGFR, malignancy type, hypertension, baseline exposure to ACEi or ARB, diuretic, PPI, or NSAID, checkpoint inhibitor type, and Charlson Comorbidity Index.

^■^Adjusted for age, sex, baseline eGFR, malignancy type, hypertension, baseline exposure to ACEi or ARB, diuretic, PPI, or NSAID, prior chemotherapy, and Charlson Comorbidity Index.

^a^Combined therapy consisted of nivolumab and ipilimumab.

### Renal outcomes and mortality

The median time between baseline creatinine measurement and last creatinine measurement was 36 (IQR 14 to 77) weeks. Persistent renal dysfunction at the end of follow-up was seen in 34 (35.4%) patients and renal recovery in 62 (64.6%) patients of all patients who had developed AKI (Table 2). The incidences of persistent renal dysfunction in patients with ICPi-AKI (40.6%) and patients with non-ICPi-AKI (32.8%) were not different (p-value 0.45). Overall mortality was high in our cohort. After a median follow-up time of 44 (IQR 22 to 89) weeks, 314 (46.4%) patients had died (mortality rate 367 per 1000 person-years). After adjustment for potential confounders, all-cause AKI was associated with a 2.13-fold (95% CI 1.58 to 2.87) increased mortality risk. Further analysis differentiating between AKI etiologies showed that patients with non-ICPi-AKI had an increased risk of mortality (HR 2.87, 95% CI 2.04 to 4.04), but patients with ICPi-AKI (definite, probable, and possible) had no higher risk of mortality (HR 1.11, 95% CI 0.64 to 1.92) (Table 4).

**Table 4:** AKI and mortality.

		Hazard ratio (95% CI)			
		Crude		Adjusted*	
No AKI	N = 580	1	(reference)	1	(reference)
AKI	N = 96	2.18	(1.62–2.93)	2.13	(1.58–2.87)
ICPi-AKI	N = 32	1.17	(0.68–2.03)	1.11	(0.64–1.92)
Non-ICPi-AKI	N = 64	2.83	(2.03–3.93)	2.87	(2.04–4.04)

AKI, acute kidney injury; ICPi-AKI, immune checkpoint inhibitor-associated AKI.

*Adjusted for age, sex, baseline eGFR, malignancy type, hypertension, baseline exposure to ACEi or ARB, diuretic, PPI, or NSAID, prior chemotherapy, checkpoint inhibitor type, and Charlson Comorbidity Index.

## Discussion

In this observational study, the overall incidence of AKI among 676 patients receiving ICPi was 14.2%. Baseline variables independently associated with AKI were a gynecologic malignancy, monotherapy with ipilimumab, and the use of a diuretic, ACE-inhibitor or ARB, or proton pump inhibitor at baseline. AKI was ICPi-associated in one third of all patients with AKI. ICPi-AKI was mostly low-grade, occurred a median of 15 weeks after ICPi initiation, and resulted in persistent renal dysfunction in approximately 40% of the patients. Although patients with all-cause AKI had a twofold increased mortality risk, ICPi-AKI was not associated with increased mortality.

### Incidence of AKI and ICPi-AKI

The incidence of AKI in our cohort was similar to the results of two other cohort studies on AKI in ICPi users by Meraz-Muñoz et al. and Seethapathy et al. [18,19]. These studies both reported an AKI incidence of 17%. With regard to ICPi-AKI, our observed incidence (4.7%) was higher than found in a meta-analysis by Cortazar et al. among a total of 3695 patients treated with ICPi in phase 2 and 3 clinical trials, where the estimated incidence of ICPi-AKI was 2.2% [15]. However, the true incidence of ICPi-AKI may have been underestimated in clinical trials, due to delay in AKI onset after ICPi initiation and inaccurate attribution of mild cases of ICPi-AKI to other etiologies. The ICPi-AKI incidence in our cohort differed from the incidences reported by Seethapathy et al. (3.0%) and Meraz-Muñoz et al. (9.7%) [18,19]. A possible explanation for the difference between the findings of Seethapathy et al. and our findings could be that Seethapathy et al. categorized AKI without a plausible etiology as “AKI of undetermined cause”, while in our study, this was recorded as possible ICPi-AKI. Both Seethapathy et al. and Meraz-Muñoz et al. only reviewed episodes of sustained AKI (i.e. AKI lasting ≥72 hours) for a potential relationship with ICPi, and episodes of transient AKI (i.e. AKI lasting <72 hours) were excluded. However, it is unlikely that this caused differences between our observed ICPi-AKI incidences, as in the study of Seethapathy et al., there were no cases of potential ICPi-AKI in patients with transient AKI, and only a minority (14%) of AKI patients in the study of Meraz-Muñoz had transient AKI. We did not differentiate sustained AKI from transient AKI, as a considerable number of patients in our cohort had intervals longer than 72 hours between creatinine measurements. These longer intervals between creatinine measurements might be explained by the low AKI stage in the majority of AKI patients.

### Risk factors for AKI

Although a gynecologic malignancy was present in only a small number of patients, this baseline characteristic was the strongest predictor for AKI in ICPi recipients. While this result has not been reported by the other cohort studies, gynecologic cancers are known to increase risk of AKI and chronic kidney disease in the general oncology population by ureteral obstruction and retroperitoneal fibrosis caused by radiotherapy [28]. Indeed, in three out of seven (42.9%) patients with a gynecologic tumor and AKI, the cause of AKI was post-renal obstruction. Secondly, monotherapy with ipilimumab was associated with increased risk of AKI. Seethapathy et al. also reported ipilimumab use to increase AKI risk (HR 1.85, 95% CI 1.05 to 3.27), however, the calculated p-value (0.21) did not reach statistical significance [19]. The meta-analysis by Cortazar et al., comparing renal toxicity among ICPi, showed that the combination of ipilimumab plus nivolumab caused higher AKI rates (4.9%) than ipilimumab alone (2.0%) [14]. Additionally, a case-control study by Cortazar et al., with 138 biopsy-confirmed ICPi-AKI patients and 276 control patients without AKI, found combined ICPi treatment, and not ipilimumab monotherapy, to be a risk factor for ICPi-AKI [14]. Thirdly, the baseline use of specific medication, i.e. diuretics, ACE-inhibitors or ARBs, and proton pump inhibitors, was also associated with increased risk of AKI. Among these drugs, only proton pump inhibitor use has been previously described to be a risk factor for development of AKI and ICPi-AKI in patients using ICPi [14,19]. Although the causal pathway remains unknown, previous studies have hypothesized that ICPi do not only induce loss of host tolerance for self-antigens, but also for exogenous agents like proton pump inhibitors [29,30]. The association between AKI and diuretics, ACE-inhibitors, and ARBs has not been previously reported in ICPi users, however, the mechanism might be similar. Additionally, these drugs may induce pre-renal AKI. Finally, our study and previous observational studies on AKI in patients using ICPi found no significant effect of female sex on the risk of AKI nor ICPi-AKI [14,18,19]. These observations contrast with results of a large meta-analysis demonstrating that the risk of developing hospital-associated AKI is significantly greater in men than in women [31]. Furthermore, animal- and clinical studies have shown that premenopausal women are relatively protected from renal disease as compared with age-matched men. However, this effect seems to disappear with advancing age and menopause, suggesting a protective role of estrogen [32–34]. As female patients in our study had a median age of 61 (IQR 49 to 71) years, they would not benefit from such a potential renoprotective estrogen effect. In addition, risk factors for AKI in the general population could be different from those in our patients, who had advanced malignancies and received anticancer therapy.

### Renal outcomes and mortality

The association between AKI and renal recovery status is relevant, as the case-control study by Cortazar et al. showed that absence of renal recovery in patients with ICPi-AKI was independently associated with increased mortality [14]. The same study reported that complete and partial renal recovery occurred in 40 and 45% of patients with ICPi-AKI, respectively. Partial renal recovery was defined as a return of creatinine to less than twice the baseline value or discharge from renal replacement therapy regardless of the creatinine value. In our cohort, with the use of different criteria of renal recovery, patients with ICPi-AKI showed renal recovery in approximately 60% at the end of follow-up. This may indicate that ICPi-AKI more often progresses to long-term impairment of renal function than initially thought.

The only other cohort study exploring the association between AKI and mortality found that AKI was not associated with increased mortality among 309 patients receiving ICPi, using Kaplan-Meier survival analysis [18]. However, the risk of mortality was not adjusted for variables potentially confounding the association between AKI and mortality (such as medication use at baseline). Furthermore, the Kaplan-Meier model does not consider time-dependency in the relationship between AKI and subsequent mortality. In our cohort, AKI was associated with a twofold increased mortality risk using a time-dependent Cox proportional hazard regression model. Increased mortality risk was not seen in patients with ICPi-AKI, but only in patients with AKI with a non-ICPi-related etiology. The increased mortality risk in patients with non-ICPi-AKI might be a reflection of concurrent problems in these patients, such as infections and tumor progression. The fact that mortality did not increase with ICPi-AKI could be related to the low AKI stages in ICPi-AKI patients (>80% had stage 1 and no patients had stage 3). An alternative theory is that occurrence of irAE, including ICPi-AKI, represents effective antitumor response and hence might improve survival. This hypothesis is suggested by recent studies showing improved progression-free and overall survival in ICPi users who develop irAE [35,36].

### Kidney biopsy

Since the American Society of Clinical Oncology recommends to forego kidney biopsy and proceed directly with immunosuppressive therapy in case of suspected ICPi-related renal toxicity [37], patients with histopathological evidence of ICPi-AKI are scarce. In our study, as in the study of Seethapathy et al. [19], biopsy was performed in only one patient with suspected ICPi-AKI, showing acute tubulointerstitial nephritis. Unfortunately, no clinical characteristics accurately differentiate between ICPi-AKI and other causes of AKI. However, some characteristics are suggestive, e.g. sterile pyuria, non-kidney irAE, and eosinophilia [26]. Therefore, we chose to use the definition and classification system of ICPi-AKI proposed by Gupta et al., which incorporates these clinical characteristics and accordingly acknowledges several gradations of diagnostic uncertainty in the absence of a kidney biopsy [26]. In addition to the recommendation to forego kidney biopsy, the American Society of Clinical Oncology advises to empirically treat an increase in creatinine ≥2 times the baseline value by interrupting ICPi therapy and to start intravenous prednisone or equivalent after exclusion of other etiologies [37]. As the majority of patients with ICPi-AKI in our cohort had stage 1 AKI (i.e. creatinine elevation <2 times the baseline value), this could explain why treatment with corticosteroids and interruption of ICPi was only initiated in less than half of the patients.

### Strengths and limitations

This study has several strengths. We were able to demonstrate the incidence of AKI in a representative population of patients using a variety of ICPi classes. Patient data were collected using a comprehensive infrastructure of databases, and determination of AKI etiology was thoroughly performed by three researchers. A strength of the Cox regression was that we used time-dependent analyses to show the association between AKI and mortality. Furthermore, to the best of our knowledge, this is the first study to provide information on the association between ICPi-AKI and mortality. This study also has limitations that need to be addressed. First, as previously discussed, ICPi-AKI was confirmed by kidney biopsy in only one patient. It is possible that other episodes of ICPi-AKI were erroneously attributed to non-ICPi-related causes and vice versa. Furthermore, as data on serum creatinine levels of patients were retrospectively collected, it is possible that AKI events were unnoticed and the incidence of AKI was thus underestimated. However, the mean number of creatinine measurements after ICPi initiation was 18 per patient per year, indicating that our population was carefully monitored. Theoretically, AKI incidence might have been overestimated by ICPi-associated myositis leading to increase of serum creatinine. This was, however, not the case in our study, since among all patients meeting AKI criteria, only one patient was diagnosed with myositis and this diagnosis was established more than six months after the initial serum creatinine elevation. Additionally, our definition of AKI did not meet all KDIGO criteria, which demand that the increase of baseline creatinine should have occurred within seven days prior to AKI onset. However, as ICPi treatment was usually managed in an outpatient setting, the majority of patients experiencing AKI did not have a creatinine measurement shortly before presenting with AKI. Another limitation of our study was the limited power in the association between several baseline variables and AKI. There were specifically low numbers of patients with gynecologic cancer and patients receiving ipilimumab monotherapy. Another possible limitation was that adding potentially related confounders to the same multiple regression model could distort the risk estimates due to collinearity. However, excluding risk factors could lead to under-correction. Therefore, we presented full models with many potential confounders to decrease confounding. A final important limitation is the fact that, due to the observational methodology of this study, no conclusions can be drawn on causal relations between risk factors and AKI and between AKI and mortality, as unmeasured confounding is possible.

In conclusion, clinicians should be aware that AKI is frequently observed in patients using ICPi and that renal function should be monitored closely after initiation of treatment, especially in patients using specific medication. In this observational cohort study, ICPi-associated AKI was not associated with increased mortality.
